# Establishment, characterization, and drug screening of low-passage patient individual non-small cell lung cancer *in vitro* models including the rare pleomorphic subentity

**DOI:** 10.3389/fonc.2023.1089681

**Published:** 2023-05-09

**Authors:** Ingo Andus, Friedrich Prall, Michael Linnebacher, Christina S. Linnebacher

**Affiliations:** ^1^ Patient Models for Precision Medicine, Department of General Surgery, University Medical Center Rostock, Rostock, Germany; ^2^ Institute of Pathology, University Medical Center Rostock, Rostock, Germany; ^3^ Molecular Oncology and Immunotherapy, Department of General Surgery, University Medical Center Rostock, Rostock, Germany

**Keywords:** patient-derived cell lines (PDC), lung tumors, non-small cell lung cancer (NSCLC), *in vitro* therapy response, patient-individual tumor models

## Abstract

**Introduction:**

For pre-clinical drug development and precision oncology research, robust cancer cell models are essential. Patient-derived models in low passages retain more genetic and phenotypic characteristics of their original tumors than conventional cancer cell lines. Subentity, individual genetics, and heterogeneity greatly influence drug sensitivity and clinical outcome.

**Materials and methods:**

Here, we report on the establishment and characterization of three patient-derived cell lines (PDCs) of different subentities of non-small cell lung cancer (NSCLC): adeno-, squamous cell, and pleomorphic carcinoma. The in-depth characterization of our PDCs included phenotype, proliferation, surface protein expression, invasion, and migration behavior as well as whole-exome and RNA sequencing. Additionally, *in vitro* drug sensitivity towards standard-of-care chemotherapeutic regimens was evaluated.

**Results:**

The pathological and molecular properties of the patients’ tumors were preserved in the PDC models HROLu22, HROLu55, and HROBML01. All cell lines expressed HLA I, while none were positive for HLA II. The epithelial cell marker CD326 and the lung tumor markers CCDC59, LYPD3, and DSG3 were also detected. The most frequently mutated genes included TP53, MXRA5, MUC16, and MUC19. Among the most overexpressed genes in tumor cells compared to normal tissue were the transcription factors HOXB9, SIM2, ZIC5, SP8, TFAP2A, FOXE1, HOXB13, and SALL4; the cancer testis antigen CT83; and the cytokine IL23A. The most downregulated genes on the RNA level encode the long non-coding RNA LANCL1-AS1, LINC00670, BANCR, and LOC100652999; the regulator of angiogenesis ANGPT4; the signaling molecules PLA2G1B and RS1; and the immune modulator SFTPD. Furthermore, neither pre-existing therapy resistances nor drug antagonistic effects could be observed.

**Conclusion:**

In summary, we successfully established three novel NSCLC PDC models from an adeno-, a squamous cell, and a pleomorphic carcinoma. Of note, NSCLC cell models of the pleomorphic subentity are very rare. The detailed characterization including molecular, morphological, and drug-sensitivity profiling makes these models valuable pre-clinical tools for drug development applications and research on precision cancer therapy. The pleomorphic model additionally enables research on a functional and cell-based level of this rare NCSLC subentity.

## 1 Introduction

According to the SEER database (https://seer.cancer.gov/csr/), lung and bronchus cancer ranks third within the category of common cancer types. The estimated number of new cases in 2022 in the US is 236,740, and the estimate for deaths is 130,180 accordingly. Thus, lung tumors account for 12% of all new cancer cases and 21% of all cancer deaths. The 5-year survival rate was 22.9% in the years 2012–2018. However, this large tumor entity is composed of a variety of subentities. In 2021, the updated “WHO Classification of Lung Tumors” was published (1). The principal components for classification remain morphology, supported by immunohistochemistry, followed by molecular techniques.

Many of the subentities are well studied, thanks to a large number and variety of available tumor models. These include small cell lung cancer and the most frequent non-small cell lung cancer (NSCLC) types: squamous cell carcinoma, adenocarcinoma, and large cell carcinoma. Pleomorphic carcinomas in contrast account for less than 1% of lung tumors (2). Published numbers go as low as 0.1% of all NSCLC (3), and to date, very few primary cell models are described in literature (4).

The goal of precision oncology is to offer highly effective treatment by an individualized therapy approach subsequent to comprehensive molecular, cellular, and functional analyses of the tumors. This approach is rapidly developing and has, especially for the molecular assessments, entered the mainstream of clinical practice. Functional analyses, however, require vital cells or better patient tumor models (5). Thus, in the era of precision oncology, patient tumor models are indispensable. Fittingly, the number and variety of patient-derived tumor models are ever growing. Currently, the most favored types for patient individual models are patient-derived cell lines (PDCs), patient-derived xenografts (PDXs), and patient-derived organoids (PDOs). The popularity of these models largely depends on level of complexity, required handling skillfulness, establishment success, and, especially in academia, costs.

Historically, the oldest models are cell lines. In 1951, the HeLa cell line was established from a patient with cervical cancer and thus became the first patient-derived tumor cell line (6). Since then, countless PDCs for most tumor entities have followed (5, 7–9). Only one decade later, in the 1960s, *in vivo* models followed suit. Engrafting tumor pieces to generate PDX models is a rather recent development (8, 10–12). The seminal description of the PDO culturing process by Clevers’ lab in the early 2000s (13–15) has revolutionized the patient-derived tumor modeling techniques.

However, as advanced as the organoid modeling system is, this model type bares a series of pitfalls or disadvantages, especially for academic research: the level of required skillfulness is much greater than for adherent cell lines, high(er)-throughput screening without elaborate equipment is barely feasible, and, last but not least, time and cost efficiency of adherent cell lines surpass organoids by far. Also, adherent cell lines, especially PDCs, possess a high level of predictability and utility in pre-clinical tumor therapy assessments (7). Thus, we here report on the establishment and characterization of three novel NSCLC PDC models: one model derived from an adenocarcinoma, one established from the brain metastasis of a squamous cell carcinoma, and the third PDC is of the very rare pleomorphic subentity. Finally, all PDC models underwent extensive morphological, molecular, and drug response assessments.

## 2 Materials and methods

### 2.1 Cell line establishment

Cell lines were established as previously published (8). Briefly, small pieces of the resection specimen, not required for diagnostic analyses, were received fresh from surgery. Single-cell suspensions were prepared by mechanic dissection of the tissue. The suspension was passed through a 100-µm cell strainer and washed with PBS. The cell pellet was resuspended in culture medium as described (8) and seeded in collagen-coated six-well plates. Continually growing cells were passaged and stocked regularly. Cells were routinely checked for absence of mycoplasma contamination.

The names of the cell lines consist of the following information: pseudonymized patient ID containing information on place of material collection (HRO = Hanse City of Rostock) and tumor entity/organ of origin (Lu = lung tumor, BML = brain metastasis of a primary lung tumor). Passage numbers are given for all experiments.

All processes involving patients and patient-derived material were approved by the ethics committee of the University Medical Center Rostock (UMR): A 2019-0187. General guidelines for working with patient material were followed; this included obtaining written consent for each patient in advance.

### 2.2 Cell culture

Cells were cultured in standard cell culture flasks using DMEM/F12 medium supplemented with 10% fetal calf serum (FCS) and 2 mM L-glutamine in a humidified CO_2_ incubator at 37°C. All cell culture media and reagents were purchased from PAN (PAN-Biotech GmbH, Aidenbach, Germany), and all culture plates and flasks were from Sarstedt (Sarstedt AG & Co. KG, Nümbrecht, Germany).

### 2.3 PDC quality control

#### 2.3.1 Mycoplasma

The absence of contaminating mycoplasma was checked on a routine basis using cell culture supernatant and the PlasmoTest™ - Mycoplasma Detection Kit (InvivoGen, San Diego, California, USA) according to the manufacturer’s recommendation.

#### 2.3.2 STR profiling

Concordance of PDCs and patient donor tissue was confirmed by short tandem repeat (STR) analysis as previously described (10). In short, DNA from PDCs and patient tissue was isolated and fragments of D5S818, D7S820, D16S539, D13S317, vWA, TPOX, THO1, CSF1PO, and Amelogenin were PCR-amplified with fluorescence-labeled primers. Subsequently, samples were size separated and analyzed by automated capillary electrophoresis (Thermo Fisher Scientific, Waltham, MA, USA).

### 2.4 Growth kinetics

Cells (2–5 × 10^4^ cells per well) were seeded in a 24-well plate and incubated for 24 h to allow attachment. One column of the 24-well plate was washed with PBS and stained with crystal violet solution every 24 h for 7 consecutive days resulting in quadruplicates for each time point. On the last day, plates were washed three times with PBS and left to dry at room temperature. After complete drying, 100 µl/well 1% sodium dodecyl sulfate solution was added, and plates were placed on a shaker for 10 min to dissolve the crystal violet. Absorbance measurements at 590 nm were performed using a Tecan Infinite 200 Pro (Tecan Group AG, Männedorf, Switzerland) plate reader. Measurements were normalized to the first time point measurement, and doubling time was calculated with GraphPad Prism 9.4.1 using the exponential growth equation for nonlinear regression.

### 2.5 Flow cytometry

Cells were harvested, washed, and resuspended in PBS, resulting in a concentration of 2 × 10^5^ cells/100 µl. The antibodies (1 µg per antibody and tube containing 100 µl of cell suspension) were added and incubated at 4°C for 30 min in the dark (for detailed information on antibodies, see **Table 1**). For measurement of stainings with anti-EGFR and -PD-L1 antibodies, cells were incubated with 1 µg cetuximab and durvalumab, respectively. For fluorescent detection, the primary antibodies were stained using an FITC-labeled anti-human IgG secondary antibody. Following antibody incubation, cells were washed three times with PBS and resuspended in 200 µl of PBS for measurement with a BD FACS Calibur (Becton, Dickinson & Co., Franklin Lakes, USA) device. Data analysis was done using FCSalyzer 0.9.22-alpha software.

**Table 1:** Antibodies used for flow cytometry.

Antigen	Conjugate	Manufacturer	Catalog no.
CD26	PE	eBioscience/Thermo Fisher Scientific Inc., Waltham, USA	12-0269-42
HLA I	APC	ImmunoTools GmbH, Friesoythe, Germany	21159036
HLA II	FITC	ImmunoTools GmbH	21279983
CD 326	APC	Miltenyi Biotec B.V. & Co. KG, Bergisch Gladbach, Germany	130091254
CD 90	FITC	Dianova GmbH, Hamburg, Germany	DIA120
LYPD3	APC	Sino Biological Europe GmbH, Düsseldorf, Germany	11836-H08H
DSG3	FITC	Cusabio, Houston, USA	CSB-PA007205YC01HU
CD27	FITC	ImmunoTools GmbH	21270273
Cetuximab (EGFR)	FITC	Merck KGaA, Darmstadt, Germany	Erbitux^®^
Durvalumab (PDL1)	FITC	AstraZeneca PLC, Cambridge, UK	Imfinzi^®^
human IgG	FITC	Bethyl Laboratories, Inc., Montgomery, USA	800-338-9579

### 2.6 Chemotherapy

Cells were seeded on a 96-well plate (1–2 × 10^4^ cells per well) in 150 µl/well standard medium and incubated for 24 h to facilitate attachment. The chemotherapeutics were added to the wells in 50 µl of standard medium yielding the desired final concentrations. For every single agent tested, detailed information can be found in **Supplementary Data 1** (**Supplementary Table 1**). After a 72-h incubation period, a second treatment cycle was initiated by the removal of old medium and the addition of new medium and chemotherapeutics. Plates were incubated for an additional 72 h. After a total of 144 h of treatment, cells were washed with PBS and stained with 50 µl/well crystal violet solution for 20 min. The crystal violet solution was then removed, and plates were washed three times with PBS. After complete drying at room temperature, 100 µl/well 1% sodium dodecyl sulfate solution was added, and plates were placed on a shaker for at least 10 min to dissolve the crystal violet. Absorbance measurements at 590 nm were performed using a Tecan Infinite 200 Pro plate reader and normalized viability was calculated in relation to untreated control samples using the following formula:


$$\text{normalized viability}=\frac{\text{OD}\left[\text{sample}\right]-\text{OD}\left[\text{blank}\right]}{\text{OD}\left[\text{living control}\right]-\text{OD}\left[\text{blank}\right]}$$

#### 2.6.1 Determination of single-agent IC_50_ value

Cells were seeded in triplicate on a 96-well plate and chemotherapy testing was performed as described above. IC_50_ values were calculated using the nonlinear regression function with a four-parameter variable slope and automatic outliner elimination with Q = 1% of GraphPad Prism 9.4.1. We repeated this for each substance at least three times independently, but with adapted concentrations at times.

### 2.7 Drug combinations and identification of additive, synergistic, or antagonistic effects

Same as for the single-agent testing, cells were seeded in a 96-well plate in standard medium. One row was used as blank control (cells incubated with PBS instead of standard medium, thus leading to cell death by starvation) and one row was used as living control (≙ untreated cells), leaving two 6 × 6 dose response matrices for drug sensitivity testing. We tested drug combinations that are typically used in clinical practice, by combining a platinum-based drug (cisplatin and carboplatin) with etoposide, vinorelbine, and paclitaxel, resulting in six paired combinations (for detailed information, please refer to **Supplementary Data 2** (**Supplementary Table 2**). Each dose–response pair experiment was repeated at least three times. Chemotherapy was performed as described above. Data obtained from the spectrometer were annotated, and normalized viability was calculated using in-house software. Synergy was calculated using the bayesynergy R package (16) and RStudio. This uses a probabilistic approach based on the Bliss independence model. The bayesynergy R package essentially describes drug interaction by comparing the zero-interaction model to the observed data using differences in normalized volume under the surface [VUS(Δ)], resulting in measures that can be directly used as percentage points of efficacy gained or lost. Values are calculated separately for antagonism VUS(Δ+) and synergism VUS(Δ−). The final synergy score is calculated by dividing VUS(Δ−) by its standard deviation (see formula below).


$$\text{Synergy Score}=\frac{\text{mean}\left(\text{VUS}\left(\text{Δ}-\right)\right)}{\text{SD}\left(\text{VUS}\left(\text{Δ}-\right)\right)}$$

### 2.8 Colony formation

Single-cell suspension was seeded at 100 cells/well in quadruplicate in a 24-well plate in 2 ml of standard medium. Outgrowing colonies were photo documented using a Primo Vert microscope and Axiocam USB camera (Zeiss, Jena, Germany).

### 2.9 Spheroid formation

After preparing a single-cell suspension, cells were seeded at 1,000 cells/well in duplicate in a cell-repellant six-well plate (Greiner AG Kremsmünster, Austria) in 5 ml of spheroid medium (CLS, Eppelheim, Germany) coated with 0.4% base agar and 0.35% top agar. Formation of spheres was checked daily and the final outcome (outgrowth or no outgrowth) was scored as positive or negative at day 14.

### 2.10 Invasion and migration

Cells were incubated in FCS-free standard medium for at least 24 h and then cells were harvested and a cell suspension of 1 × 10^3^ cells/µl was prepared in FCS-free standard medium. A 24-well plate with TC inserts (Greiner) was prepared and 500 µl of the cell suspension (≙ 5 × 10^5^ cells) was added to each insert. For the invasion assay, inserts were coated with Matrigel^®^ Basement Membrane Matrix (Corning Inc., Corning, New York, USA) prior to the addition of the cell suspension. Then, 750 µl of 10% FCS containing medium was added to the lower wells. After incubation for 72 h at 37°C and 5% CO_2_, the medium was removed, and inserts were washed twice with PBS. Finally, 1 ml of 0.2% crystal violet solution was added to each insert for cell staining. After 15 min of incubation, removal of the staining solution, and three washing steps with PBS, the non-invasive cells on the upper side of the insert were scraped off with a cotton swab. The addition of 0.75 ml of 1% SDS solution and 15 min incubation on a shaker led to solvation of crystal violet. The inserts were removed, the plate was placed in the Tecan reader, and absorbance was measured (measurement: 570 nm; reference: 620 nm). Measurements were normalized to the HROC24 cell line (17). For data analysis, again, the GraphPad Prism software was used.

### 2.11 Wound healing assay

Cells were seeded on six-well plates and allowed to grow until full confluency was reached. After starving cells for 24 h with FCS-free standard medium, a scratch was performed using a standard 200-µl pipette tip. Photos were taken with the Primo Vert microscope and Axiocam USB camera at different time periods to account for the different wound closure rates. For each cell line, the assay was done at least three times and the mean wound closure rate and standard deviation was calculated. Photos were analyzed using ImageJ 1.53 and wound healing size tool (18). Wound closure rate in µm/h was calculated in Microsoft Excel by finding the beginning $\left(t_{0}\right)\;$ and end point $\left(t_{1}\right)$ of the linear migration phase, calculating the difference in wound width during linear migration and dividing the difference by time in hours.


$$\text{wound closure rate}=\;\frac{\text{wound width }t_{0}\left(\text{µm}\right)-\text{wound width }t_{1}\;\left(\text{µm}\right)\;}{\text{time }\left(\text{h}\right)}$$

For each cell line, the assay was done at least three times and the mean wound closure rate and standard deviation were calculated.

### 2.12 DNA extraction and next-generation sequencing by whole exome sequencing

#### 2.12.1 DNA isolation

DNA from cell pellets was extracted using the Promega Wizard^®^ Genomic DNA Purification Kit and DNA from tissue was extracted using the Precellys Tissue DNA Kit (PeqLab by VWR, Darmstadt, Germany). Successful DNA isolation was confirmed by measuring DNA concentration with Nano-Drop (Thermo Scientific™, Waltham, Massachusetts, USA).

#### 2.12.2 DNA NGS sequencing and bioinformatic analysis

Library preparation and sequencing was done by an external facility (IKMB, Kiel, Germany). Bioinformatics were partly done on the Galaxy Europe platform (19, 20). After initial quality control with FastQC, reads were trimmed by removing adapter sequences and low-quality reads using Trimmomatic (21). BWA-MEM (22) was used for read mapping to the reference genome GRCh38 (December 2013). Mapped reads were then filtered with BAMtools (23) in order to only keep reads where both reads have mapped to the reference genome, with a minimum mapping quality of 1. Duplicate reads were removed with the RmDup function from SAMtools (24) and indels were left aligned with the leftalign utility from FreeBayes (25). For samples with matched tumor–normal pair somatic variant, calling was done with VarScan Somatic (26) by setting the estimated tumor purity to 50% for tissue samples and 100% for cell culture and normal tissue samples. The *p*-value threshold for calling variants was set to 0.99 and the *p*-value threshold for calling somatic variants was set to 0.05. The resulting variants were then filtered using vcflib (27) and bcftools view (24) to retain only variants that have passed previous filters, are marked as somatic variants by VarScan, and have a somatic *p*-value of< 0.05. For samples without matched normal tissue samples, GATK 4.2.6.1 Mutec2 (28) was used in tumor-only mode with standard settings for somatic variant calling. In order to reduce false-positive somatic calls, we used the gnomAD (29) database as the common germline variant database for Mutec2 to identify possible germline variants better. The resulting VCF files were then converted with vcf2maf (30) and annotated by the included VEP (31) function.

For mutational signature analysis, we used the maftools (32) R package. The analysis was done with all somatic mutations including introns and synonymous mutations for each of the cell lines.

For the oncoplot visualization, in order to pick 30 mutations that could be relevant, we first included genes where multiple samples have somatic mutations. All remaining mutations from the matched tumor–normal paired samples were annotated with the Catalogue of Somatic Mutation In Cancer (COSMIC) gene (33) database using OpenCRAVAT (34) and sorted by frequency reported in the database. The most frequently mutated genes were included in the final oncoplot by using maftools (32) oncoplot function.

Tumor mutational burden (TMB) calculation was done using maftools by dividing the total number of non-synonymous coding somatic mutations by the size of the human exome [30 MB (35)].

### 2.13 RNA extraction and NGS RNA sequencing

#### 2.13.1 RNA isolation

Total RNA from cell lines was isolated by using the EurX RNA Purification Kit. Total RNA from tissue was extracted using the Precellys Tissue RNA Kit (PeqLab by VWR).

#### 2.13.2 RNA NGS sequencing and bioinformatic analysis

Sequencing was done by an external facility (IKMB). After quality control with FastQC, raw reads were trimmed using Cutadapt (36) removing low-quality reads (quality cutoff = 20) and adapter content. After alignment to the reference genome hg38 with HISAT2 (37), gene expression was measured with featureCounts (38). Differential expression analysis was then done with DESeq2. The heatmap was created by including differentially expressed genes from DESeq2 with an adjusted *p*-value< 0.01 and selecting the top 20 most significantly up- and downregulated genes.

### 2.14 Histology

Per cell line, 5 × 10^6^ cells were harvested by scraping, resuspended in PBS, and embedded in paraffin, and 4-µm sections were stained by applying the same SOP for diagnostic immunohistochemistry assessments. The following reagents/antibodies were used for staining: hematoxylin and eosin for H&E, clone 22C3 for PD-L1, polyclonal AE1/3 for pan-cytokeratin, and clone Ber-EP4 for Ep-CAM.

## 3 Results

### 3.1 Clinical and patient information

Three patients operated on at the UMR in the years 2009–2020 and enrolled in the BioBank Rostock (BBR) presented with either primary lung tumors or brain metastases (see **Table 2**). Patient HROBML01 was a male patient who presented with a metastatic lung tumor in the brain at age 67 and had no smoking habit. Patient HROLu22 was a female patient who presented with a primary tumor of the lung at age 64 and also did not smoke. Patient HROLu55 was a male patient who presented with a primary lung tumor at age 48 and was, at least at times, a heavy smoker.

**Table 2:** Overview of patient information.

	HROLu22	HROLu55	HROBML01
Gender	Female	Male	Male
Age at diagnosis	64 years	48 years	67 years
Diagnosis	Primary lung tumor	Primary lung tumor	Brain metastasis
Smoking habit	No	Yes	No

Routine diagnostic procedures included histological assessment of the tumor tissue, determination of the subentity, TNM classification, and grading. The primary lung tumors were further analyzed for TTF1 and PD-L1 protein expression and an NGS Illumina focus panel assessment was performed (see **Table 3**, and for detailed information on the Illumina focus panel, see **Supplementary Data 3**). The compiled data for the tumor of patient HROLu22 reveal a primary adenocarcinoma of the lung grade 3 with no nodal or distant metastases. Tumor cells stained positive for both TTF1 and PD-L1. The detailed molecular profiling revealed a complex mutation in the EGF receptor (EGFR). Assessments of patient HROLu55’s tumor revealed a rare pleomorphic cell tumor of the lung. The tumor was graded as G3/4 and distant lymph nodes had already been infiltrated with tumor cells. While the majority of tumor cells stained positive for PD-L1, no TTF1 protein could be detected. Tumor HROBML01 was classified as a brain metastasis of a primary squamous cell carcinoma of the lung. The origin of derived PDC models from the NSCLC patients was ensured by confirming matching STR profiles of the patients with respective PDCs (**Table 4**).

**Table 3:** Routine diagnostic histological and molecular pathological tumor assessment.

	HROLu22	HROLu55	HROBML01
Subentity	Adenocarcinoma	Pleomorphic tumor	Squamous cell carcinoma
Tumor classification	G3 pT2a pN0 cM0	G3/4 pT4 pN2 cM0	Not analyzed
TTF1 protein expression	Positive	Negative	Not analyzed
PD-L1 protein expression	Positive	Positive	Not analyzed
Illumina focus panel	EGFR: E746 T751delinsV-Mutation (Exon19)	No mutations detected	Not analyzed

**Table 4:** STR profiles.

		D5S818		D13S317		D7S820		D16S539		vWA		TH01		TPOX		CSF1 P0		Amelogenin
**HROBML01**	Tumor	12	13	10		10	11	11	12	17		7		8		12		f
	PDC	12	13	10		10	11	11	12	17		7		8		12		f
**HROLu22**	Normal	10	12	11	12	8	10	12	14	17	18	7	9	8		10	11	f
	Tumor	10	12	11	12	8	10	12	14	17	18	7	9	8		10	11	f
	PDC	10	12	11		8	10	12	14	17	18	7	9	8		10	11	f
**HROLu55**	Normal	11	12	11	15	8	11	11	13	16	18	7	9	8	11	12		m
	Tumor	11	12	11	15	8	11	11	13	16	18	7	9	8	11	12		m
	PDC	11	12	11		8	11		13	16	18	7	9	8	11	12		m

Normal: STR profile of normal lung tissue, Tumor: STR profile of NSCLC tissue, PDC: STR profile of PDC model.

### 3.2 Tumor model characteristics

**Figure 1:**
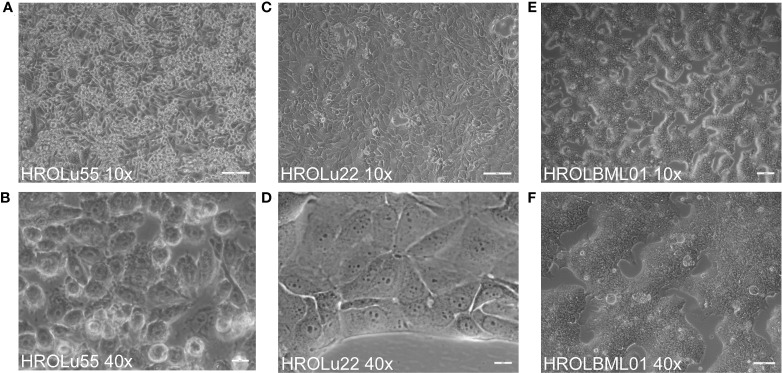
Cell morphology. Depicted are light microscopy images of the cell lines HROLu55 (left, **A**, **B**), HROLu22 (middle, **C**, **D**), and HROBML01 (right **E**, **F**) at 10-fold (top row) and 40-fold (bottom row) magnification.

The cells of HROBML01 and HROLu22 grow as tumor islands, and the cells possess a morphology frequently associated with epithelial tumor (i.e., cobblestone-like) cells. The cells of HROLu55 start of as large(r) cells, which reduce their size according to the space available and finally form a 100% confluent monolayer consisting of small cells in the end. Micro-photographic images of all three cell lines can be found in **Figure 1**.

Cell doubling times were between 3 and 5 days. The highest doubling time with 69.31 h (ranging from 57.60 h to 87.00 h; 95% CI) was calculated for HROBML01, closely followed by HROLu55 with 72.06 h (61.85 h to 86.32 h; 95% CI). The lowest proliferation was observed for HROLu22 with a doubling time surpassing 100 h: 101.6 h (91.77 h to 113.7 h; 95% CI).

### 3.3 Histology

**Figure 2:**
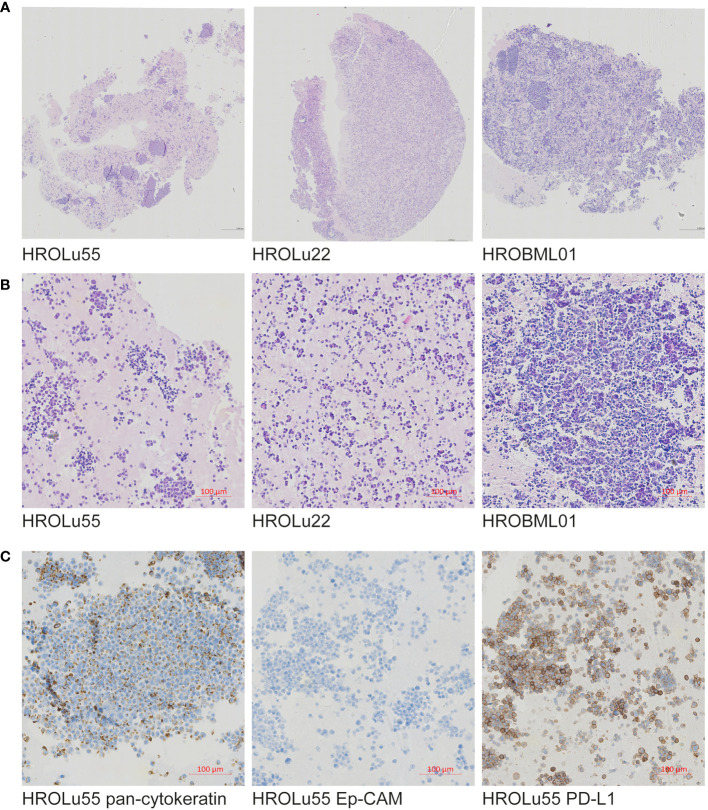
Immunohistochemistry. Presented are scans of slides from FFPE embedded cells of the cell lines HROLu22, HROLu55, and HROBML01. The top row **(A)** shows H&E overview staining for all three cell lines [HROLu55 (left), HROLu22 (middle), and HROBML01 (right)]. The middle row **(B)** shows part of the H&E staining for all three cell lines [HROLu55 (left), HROLu22 (middle), and HROBML01 (right)]. The bottom row **(C)** shows protein staining against pan-cytokeratin (left), Ep-CAM (middle), and PD-L1 (right).

Paraffin-embedded immunohistochemical assessments performed by an expert pathologist (FP) of the three cell lines (see **Figure 2**) confirmed in H&E overview staining that the cell line HROBML01 is most likely derived from a squamous cell type, HROLu22 from an adenocarcinoma, and HROLu55 from a pleomorphic carcinoma. Additionally, the embedded cells of HROLu55 stained positive for pan-cytokeratin and Ep-CAM as well as PD-L1, which further supports the pleomorphic subentity of the cell line HROLu55 as well.

### 3.4 Flow cytometry

**Figure 3:**
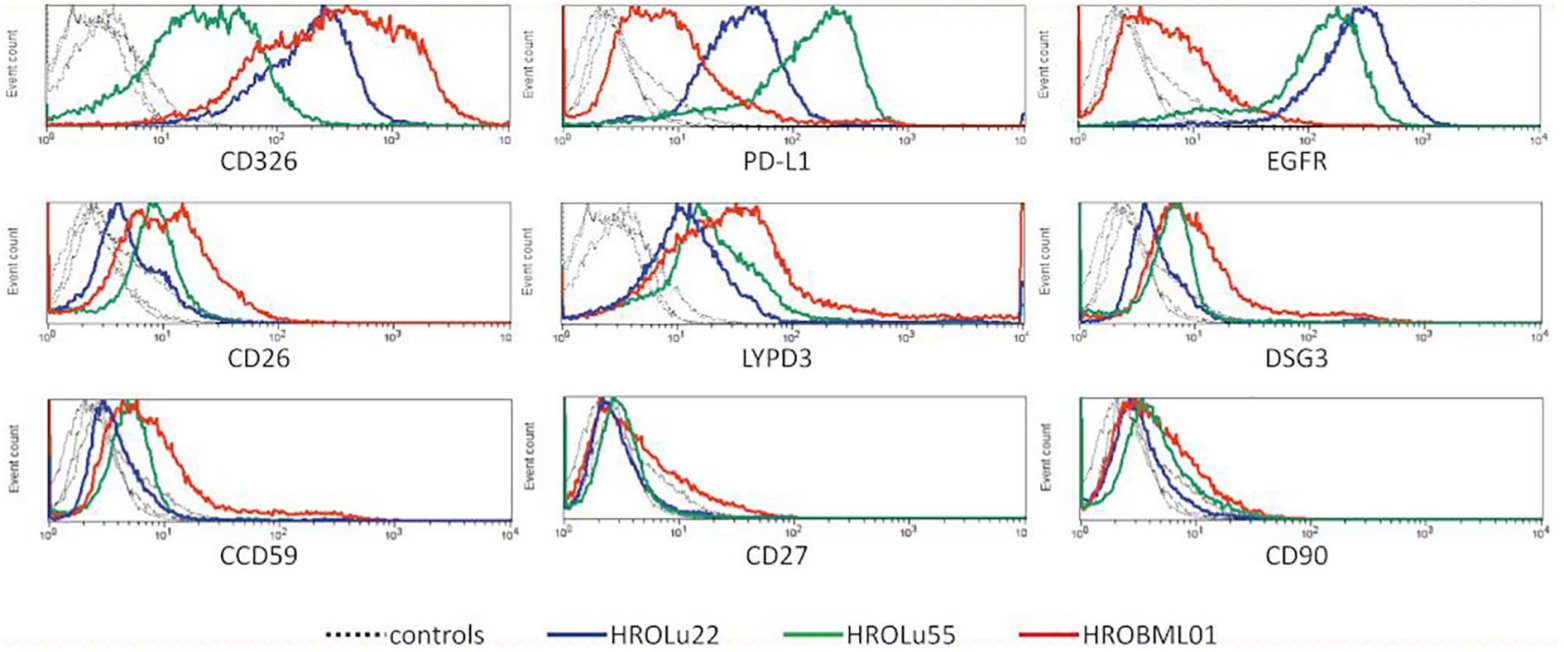
Flow cytometry. Histogram overlays of unstained controls (dotted lines for all three cell lines) and measurements for HROLu55 (green), HROLu22 (blue), and HROBML01 (red) for the epitopes CD326, PD-L1, EGFR, CD26, LYPD3, DSG3, CCD59, CD27, and CD90 are shown.

After confirmation that the cell lines represent the subentities of the tumors they were derived from, the expression of several surface markers was assessed by flow cytometry (**Figure 3**). All three PDCs expressed HLA I and lacked HLA II expression (data not shown). Because high levels of CD326 (Ep-CAM) were considered as a marker for the epithelial origin of cells, the absence of CD90 as a marker for fibroblast cells, and expression of proteins as a marker for lung tumors, LYPD3, DSG3, and CCD59 (39) were analyzed. All cells expressed CD326, were negative for CD90, and had varying degrees of the lung tumor markers. Additionally, the presence of the immune checkpoint protein PD-L1 and the growth receptor EGFR was analyzed. The cell lines derived from the primary lung tumors were strongly positive for PD-L1 and EGFR. The brain metastasis cell line HROBML01 only showed weak staining for both markers.

### 3.5 DNA sequencing

In addition to the focus panel sequencing, which was performed during routine pathological assessment for the tumors of patients HROLu22 and HROLu55, we performed whole exome sequencing (WES) analyses, comparing the cell lines with the original patient tumors for all three models. For patients HROLu22 and HROLu55, tumor adjacent normal tissue was assessed in parallel. Due to the fact that HROBML01 was derived from a brain metastasis, no normal brain tissue was available.

**Figure 4:**
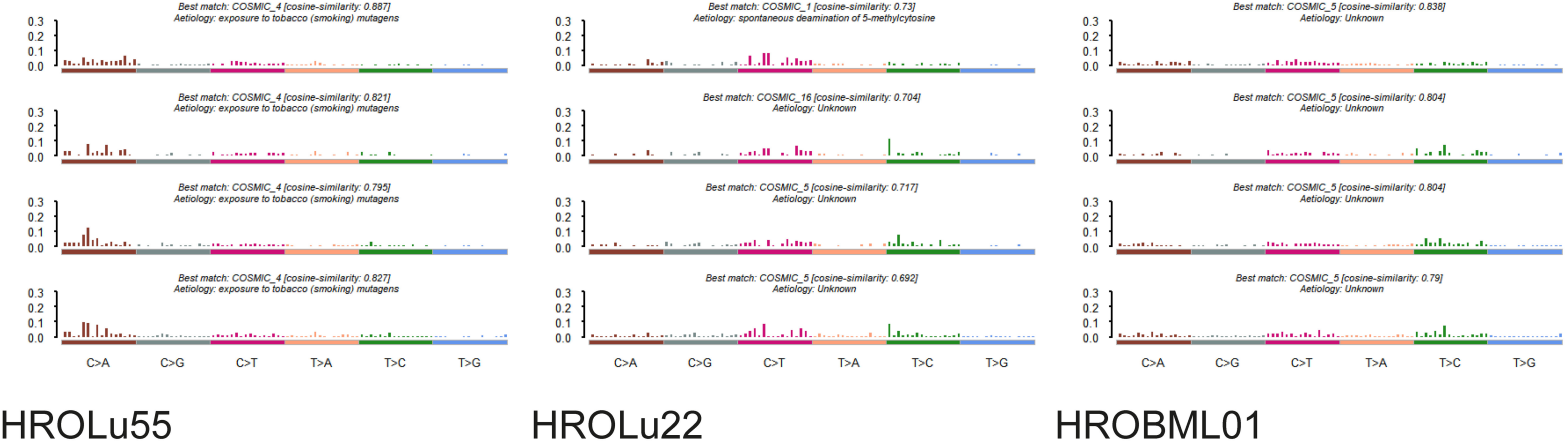
Mutational signatures. Represented are the base substitutional distributions and most probable associations according to COSMIC (https://cancer.sanger.ac.uk/signatures/) for the cell lines HROLu55 (left), HROLu22 (middle), and HROBML01 (right).

The mutational signature analysis of HROLu55 shows high similarity to cosmic signature 4, which is consistent with the mutational pattern caused by exposure to tobacco smoke (**Figure 4**). No such clear association with a mutational pattern could be observed for either HROLu22 or HROBML01 (see **Figure 4**). However, HROLu22 shows similarities to signatures 1, 5, and 16. Signature 1 is believed to be related to an endogenous mutational process, signature 5 is not yet associated with an underlying mechanism but is often observed in lung adenocarcinomas (40), and signature 16 does not have any described underlying mechanism so far. HROBML01 shows again the highest similarity to signature 5.

**Figure 5:**
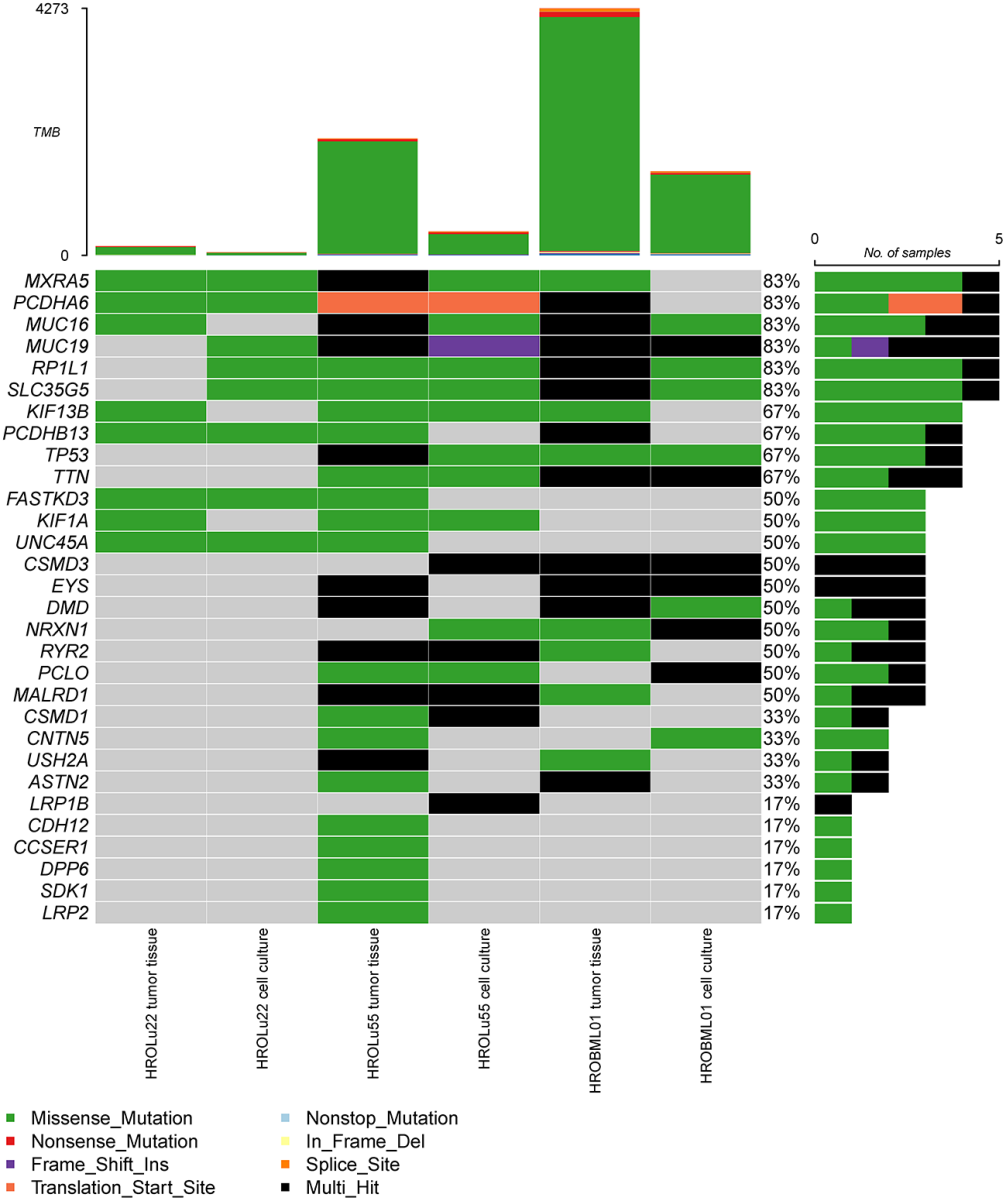
Most frequent mutations. The oncoplot shows the most frequent mutations and types across all samples for tumor tissues and cell cultures of commonly mutated genes present in the COSMIC gene database.

In a second step, we identified the top 30 mutated genes by the WES approach across all three tumor tissues and cell lines (see **Figure 5**). Among these, common general cancer mutations in the genes TP53 and MUC16 (41) and lung cancer-associated mutations in the genes MXRA5 (42, 43) and MUC19 (44) could be observed. The genes MXRA5 and MUC16 were mutated in all three tumors. However, the mutation in MXRA5 could not be detected in the cell line HROBML01 and the MUC16 mutation was not detected in the cell line HROLu22. Mutations in the gene TP53 could be observed for both tumors of patients HROLu55 and HROBML01 as well as their cell line counterparts.

**Figure 6:**
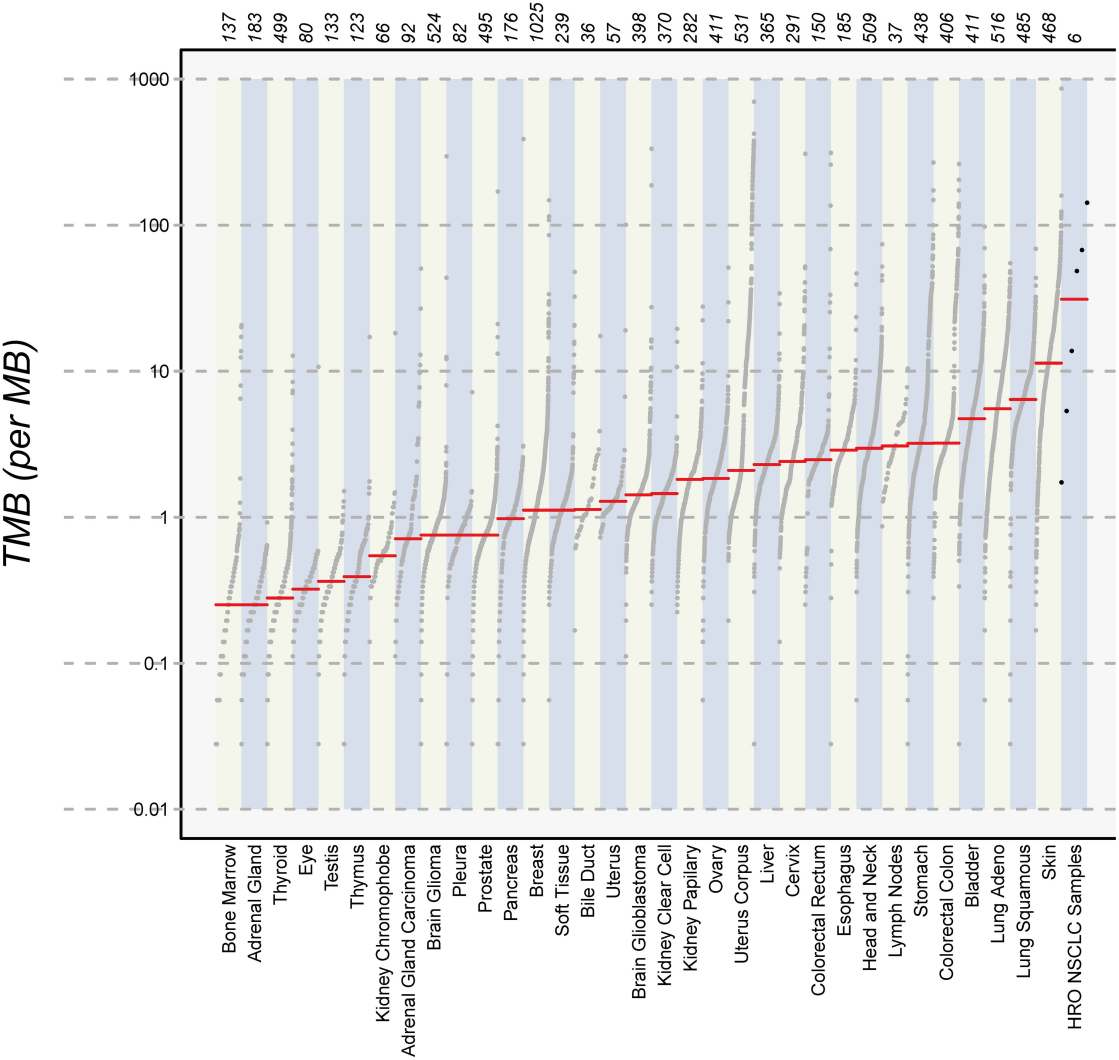
Number of mutations. The images show the extent of TMB for the HRO NSCLC samples, encompassing data from both tumor tissues and cell lines in comparison to TMB values of other cancer types recorded in the TCGA database.

Finally, we calculated the TMB for all cancer samples (tissues and cell lines). The tumor tissue always had a higher TMB than the corresponding cell line (see **Table 5**). HROBML01 presented with the highest TMB for both tumor tissue and cell line. In total, all three tumors and cell lines have a comparably high TMB (see **Figure 6**).

**Table 5:** Number of somatic mutations and TMB.

Sample		Total somatic mutations	TMB (per MB)	log TMB (per MB)
HROLu22	Cell culture	52	1.73	0.23
HROLu22	Tumor tissue	160	5.33	0.72
HROLu55	Cell culture	413	13.76	1.13
HROLu55	Tumor tissue	1,454	48.46	1.68
HROBML01	Cell culture	2,025	67.50	1.82
HROBML01	Tumor tissue	4,273	142.43	2.15

### 3.6 RNA sequencing

In addition to the WES analyses, matched RNA expression levels were assessed by RNA sequencing of the same tissue piece or cell pellet, respectively. Principal component analysis (PCA) for quality control revealed clustering of the normal tissue, the tumor tissue of HROLu22 and HROLu55 with their cell line counterparts, and a third cluster of tumor tissue and cell line for HROBML01 (see **Supplementary Data 4** consisting of **Supplementary Figures 2**, **3**). Further “sample-to-sample distance” calculations revealed that the normal tissues of HROLu22 and HROLu55 are (very) close (see **Supplementary Data 4**). This confirms the QC assessment of the PCA.

**Figure 7:**
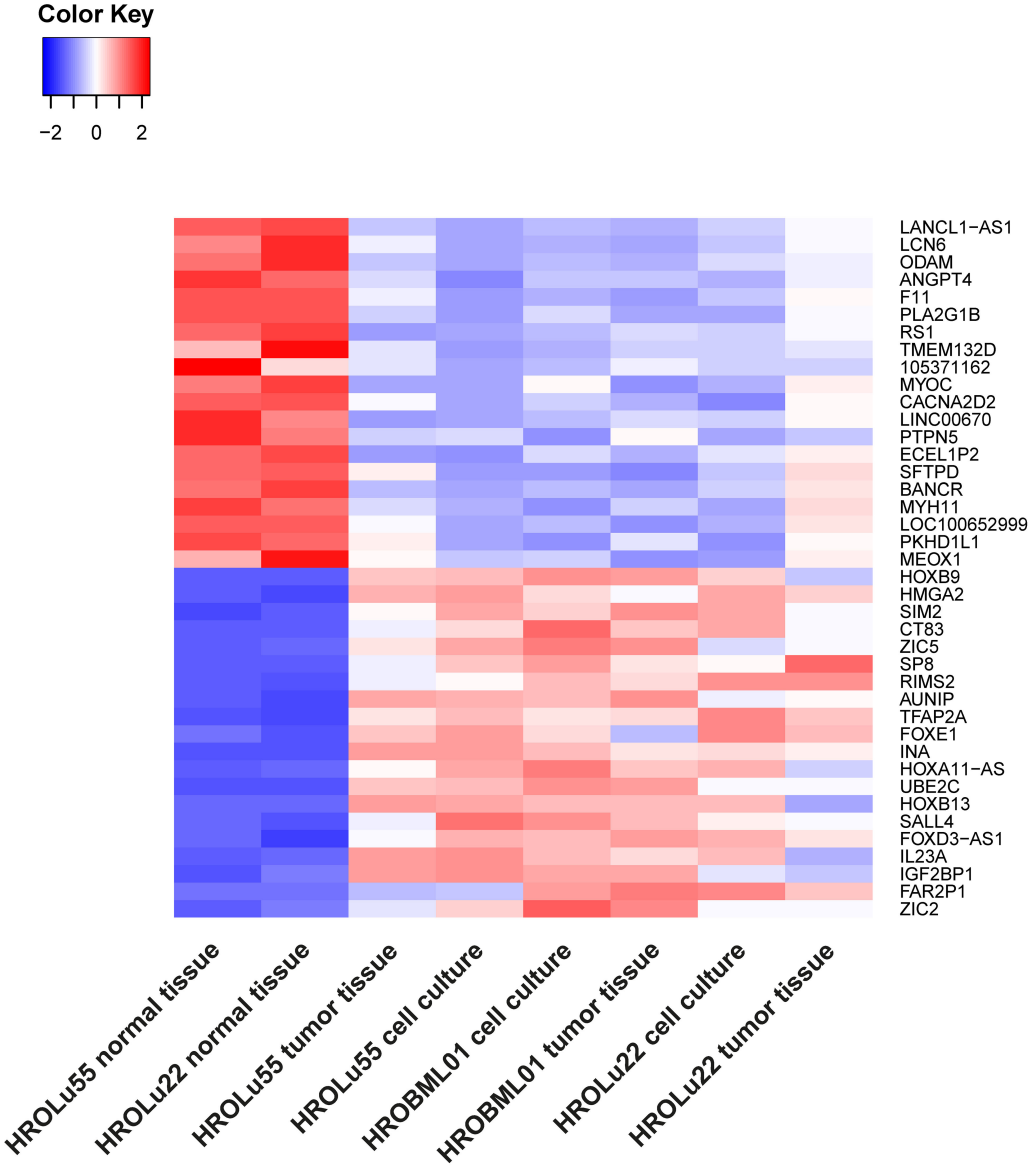
Differential RNA expression. In this graphic, the 20 most upregulated (red) and downregulated (blue) genes in tumor tissues and cell lines (on RNA level) in comparison to the corresponding normal lung tissue from the same patients with an adjusted *p*-value of< 0.01 are given.

Thus, the 20 most up- and downregulated genes on RNA expression level were identified (**Figure 7**). The difference in expression level of normal to tumor tissue and cell lines is at least log2. Among the most overexpressed genes in tumor cells compared to normal tissue are genes coding for transcription factors (HOXB9, SIM2, ZIC5, SP8, TFAP2A, FOXE1, HOXB13, and SALL4), cancer testis antigens (CT83), and cytokines activating regulatory Th17 cells (IL23A). The most downregulated genes on the RNA level code for long non-coding RNA (lncRNA; LANCL1-AS1, LINC00670, BANCR, and LOC100652999) and proteins involved in the regulation of angiogenesis (ANGPT4), signaling cascades (PLA2G1B and RS1), and immune response (SFTPD).

### 3.7 Invasion, migration, and wound healing activity

**Figure 8:**
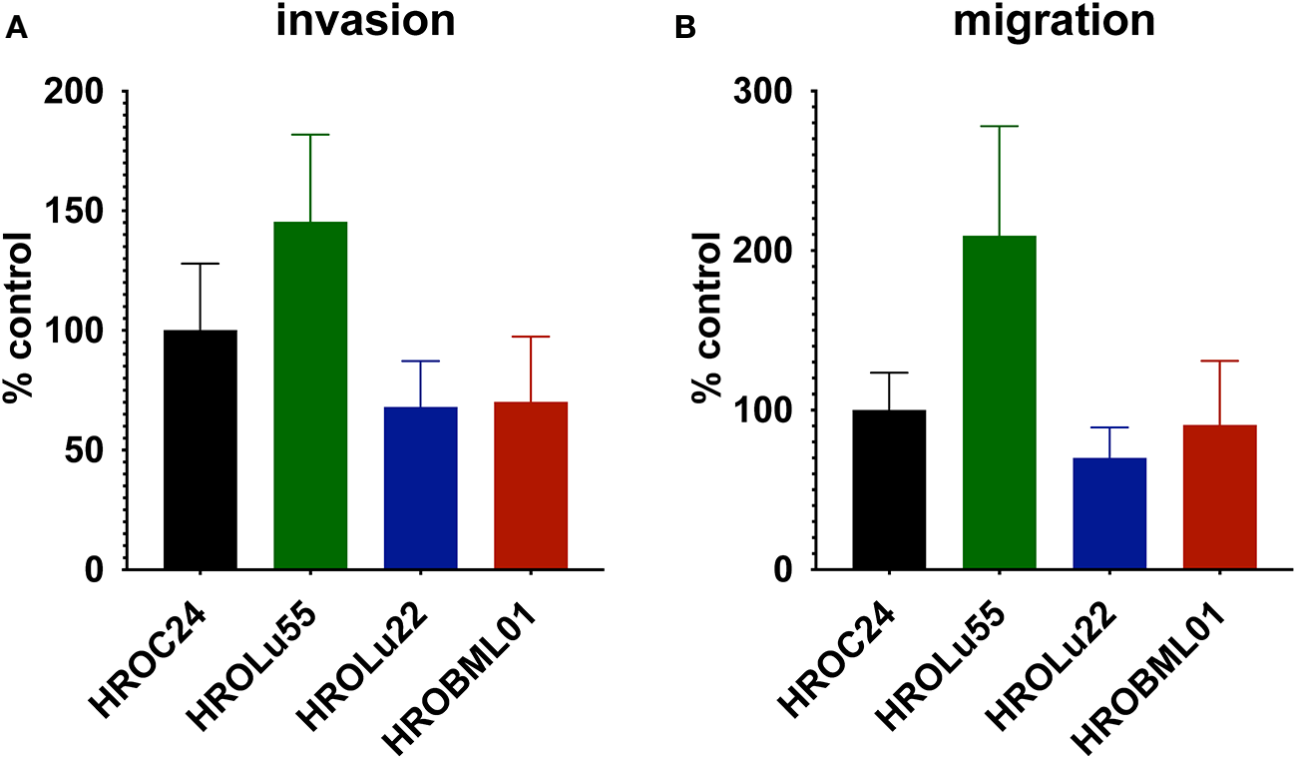
Invasion and migration. The bar graph shows the percentage of cell invasion **(A)** and cell migration **(B)** for the cell lines HROLu55 (green), HROLu22 (blue), and HROBML01 (red) in comparison to highly invasive and migration active cells HROC24 (17).

A common feature of cancer cells that serves as a unit to measure the degree of aggressiveness is invasion and migration. Thus, the invasion and migration potential of the three cell lines was assessed in classical transwell assays. In comparison to the reference HROC24, the cells of cell line HROLu55 possessed a higher capacity for invasion and migration. The cells of HROLu22 and HROBML01 were comparable to the reference with regard to both invasion and migration (**Table 6** and **Figure 8**).

**Table 6:** Invasion and migration.

		HROC24	HROLu55	HROLu22	HROBML01
Invasion (% cntrl)	Mean %	100.2	145.4	68.0	70.2
	Std. deviation	27.8	36.5	19.2	27.2
Migration (% cntrl)	Mean %	100.0	209.2	70.0	90.6
	Std. deviation	23.3	68.8	19.1	40.2

**Figure 9:**
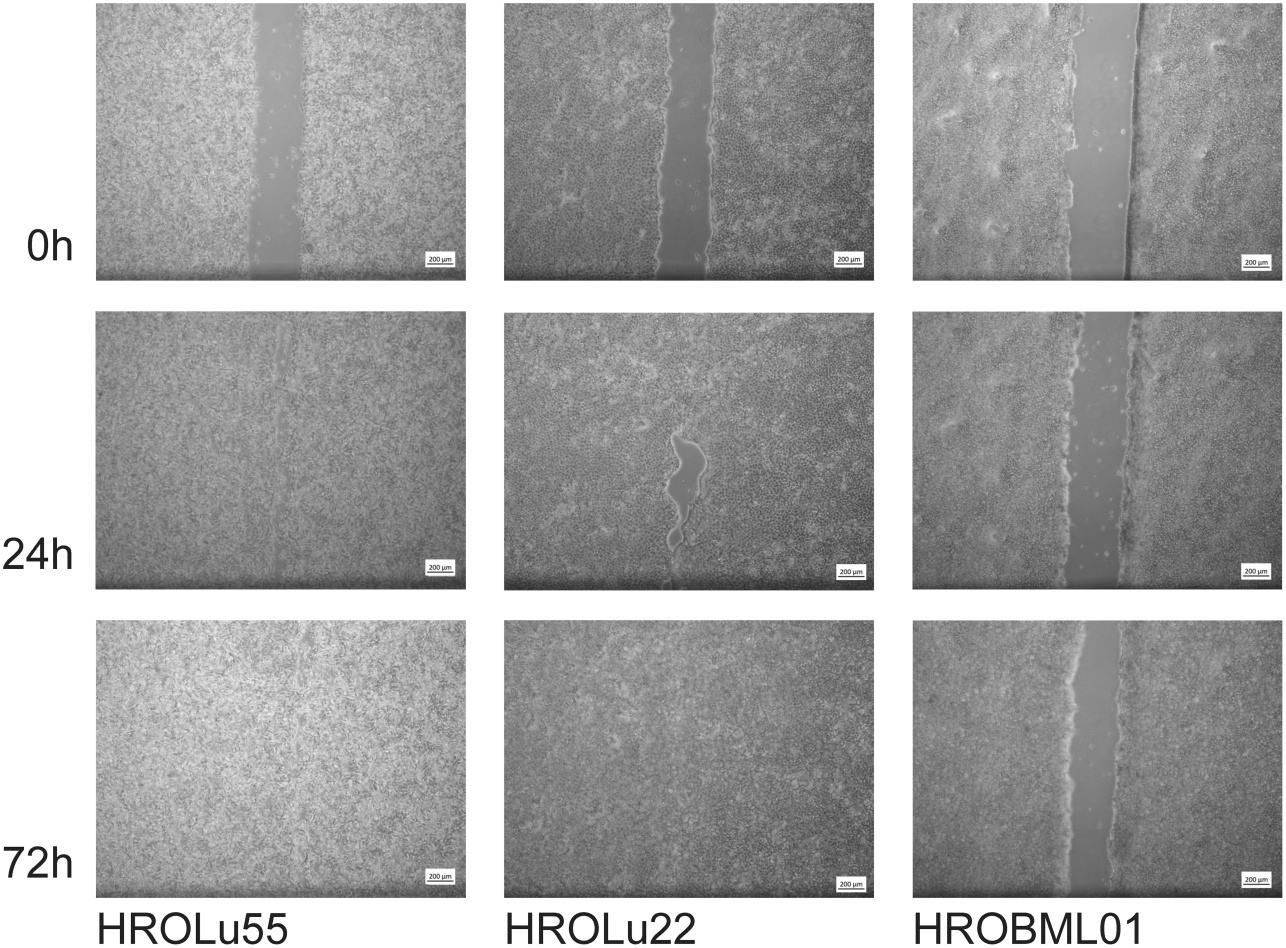
Scratch assay. Light microsopy images at 0 h, 24 h, and 72 h of scarring represent the time necessary for wound closure and thus thespeed of wound healing.

These properties of higher invasion and migration capacity resulted in faster wound closure rates (**Table 7** and **Figure 9**) for HROLu55. These cells are capable of a rapid wound closure (25.21 µm/h). Cells of HROLu22 were 8.99 µm/h slower than cells of HROLu55 but substantially faster than HROBML01. The cell line HROBML01 shows a very slow closure rate (1.23 µm/h). Unfortunately, the results for HROBML01 are inconclusive since cells tend to detach from the culture flask during or after wound infliction.

**Table 7:** Wound healing.

Closure rate (µm/h)	HROLu55	HROLu22	HROBML01
Mean	25.21	8.99	1.22
Std. deviation	10.18	2.36	0.73

### 3.8 Colony and spheroid formation

**Figure 10:**
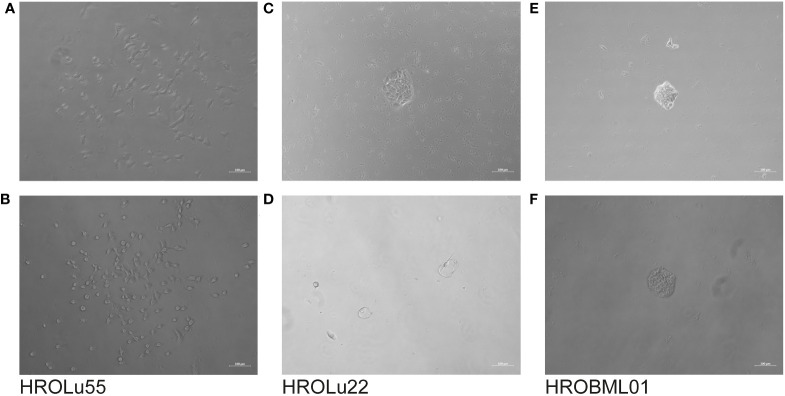
Colony formation. The light microscopy images show the progress of colony formation for the cell lines HROLu55 (left, **A, B**), HROLu22 (middle, **C, D**), and HROBML01 (right, **E, F**). The images are representative of four wells each.

Further properties associated with tumorigenicity are the capacity to form colonies starting with very few tumor cells (≈100 cells) and three-dimensional spheroids (**Figure 10**). Classical epithelial tumor cell colony formation was observed for HROBML01 and HROLu22. Cells of the cell line HROLu55 also grew in colony-like formations; however, these resemble more a loose accumulation of cells without direct cell-to-cell interaction.

Spheroid formation was observed after 7–10 days for all three cell lines. The earliest onset and most pronounced spheroid formation was observed for HROLu55 (data not shown).

### 3.9 *In vitro* drug response

**Figure 11:**
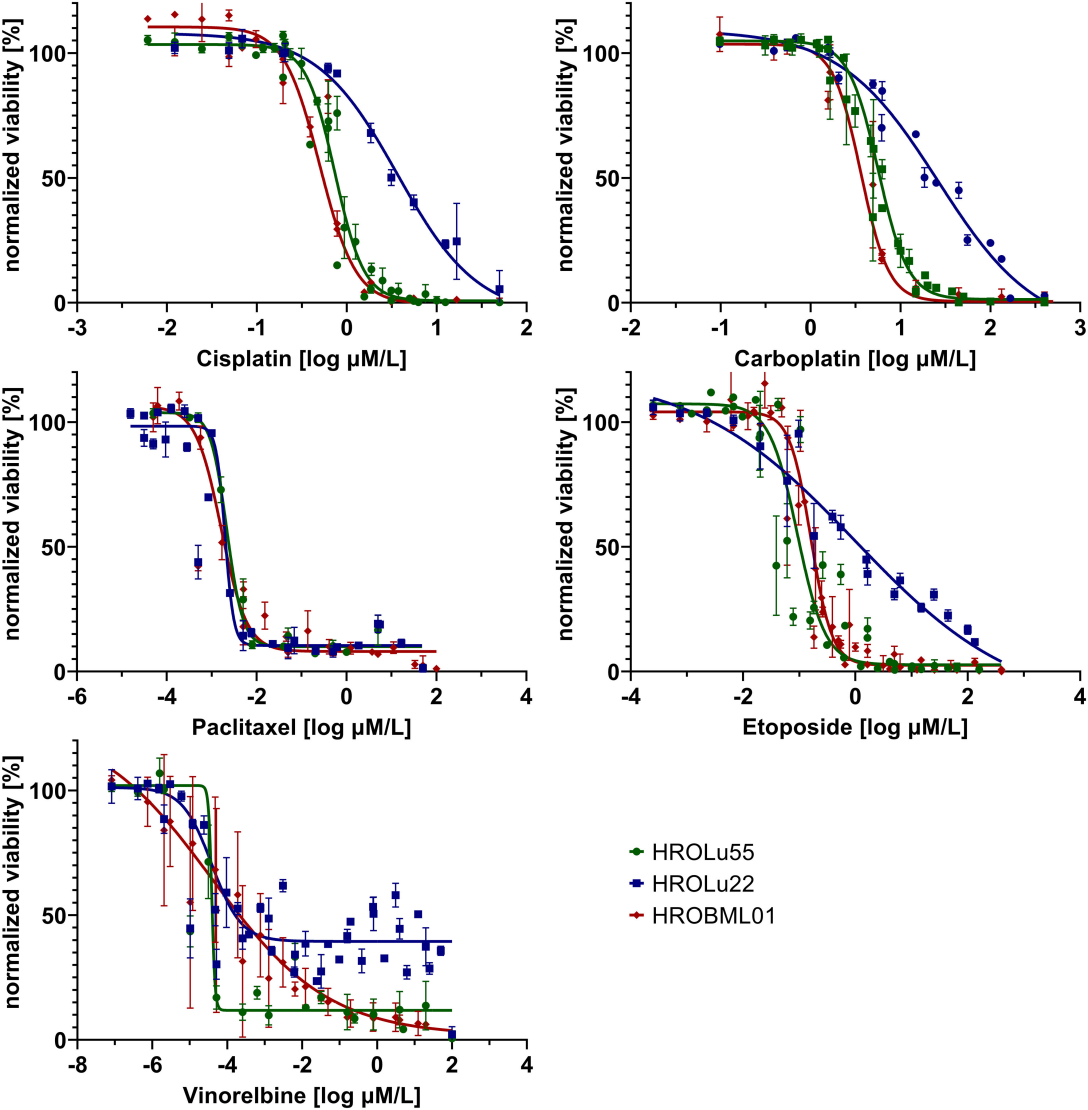
Dose response. Dose kinetic response of single-agent treatments of the cell lines HROLu55 (green), HROLu22 (blue), and HROBML01 (red) for 144 h for the agents: cisplatin, carboplatin, paclitaxel, etoposide, and vinorelbine are plotted as normalized viability with standard deviation over dose range in log µM.

Adherent 2D cell lines, especially in the NCI-60 panel, have a long-standing tradition as tools for *in vitro* response testing (45). In particular, patient-derived cell lines possess great potential for highly accurate predictability (7). Thus, response to a broad variety of therapeutics commonly administered for treatment of lung tumors was determined in dose-kinetic analyses (**Figure 11**). All IC_50_ value calculations are in the range typically found in clinical settings (**Table 8**). Thus, pre-existing drug resistance for all three cell lines and tested reagents is unlikely. HROLu22 tended to be least sensitive towards treatment with cisplatin, carboplatin, and etoposide. Response to vinorelbine in HROLu22 cells plateaued over a (wide) range of concentrations until viability finally decreased to 0.

**Table 8:** IC_50_ values.

IC_50_ (in µM)	HROLu55	HROLu22	HROBML01
Cisplatin	0.737	3.659	0.501
Carboplatin	5.657	26.160	3.623
Paclitaxel	2.169 × 10^−3^	1.978 × 10^−3^	1.632 × 10^−3^
Etoposide	0.093	0.987	0.162
Vinorelbine	2.615 × 10^−5^	3.596 × 10^−5^	15.3 × 10^−5^

### 3.10 Drug combinations and additive, synergistic, or antagonistic effects

**Figure 12:**
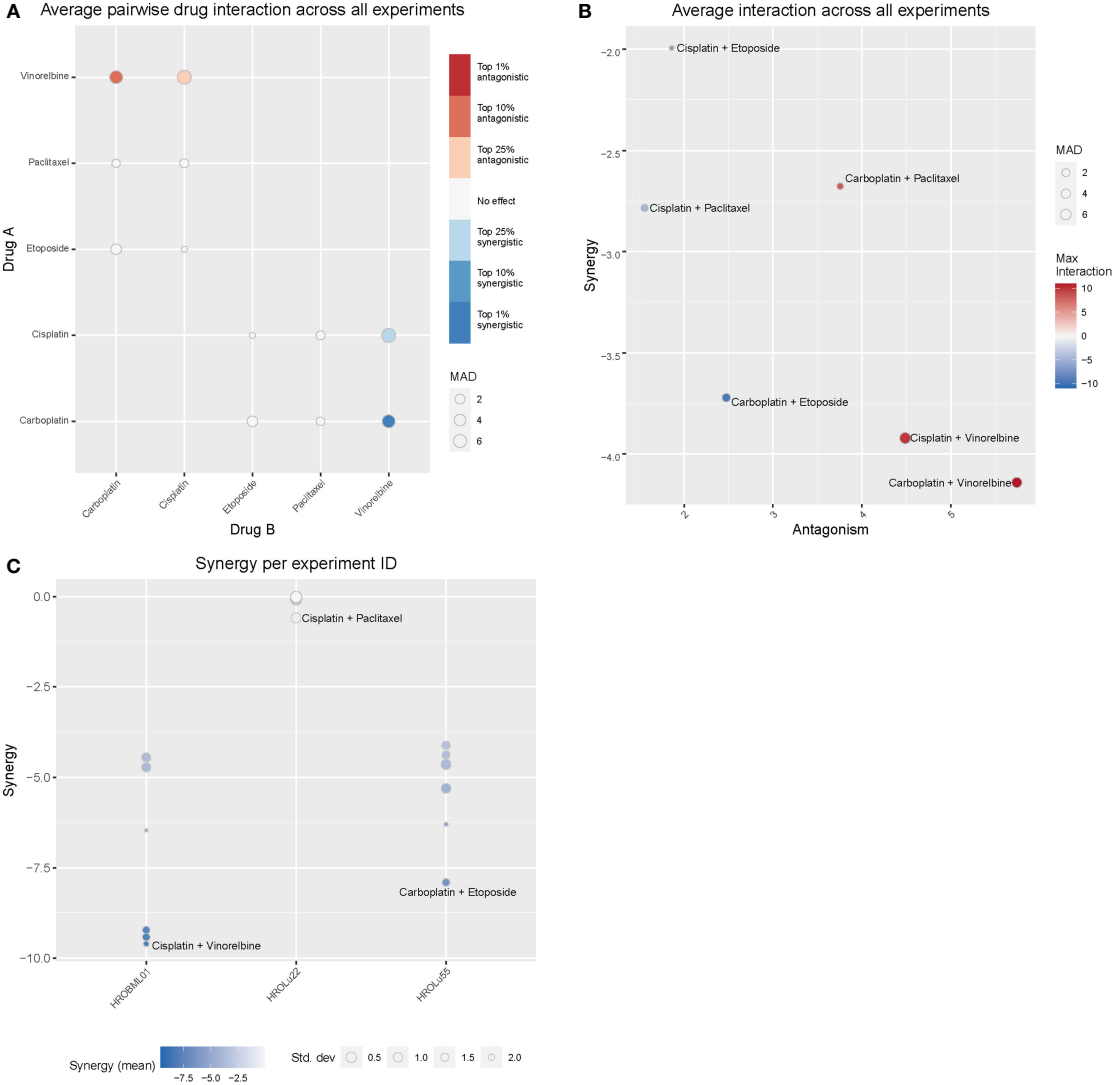
Drug combination treatments. Bliss independence-based synergy/antagonism scores are given for **(A)** the average pairwise drug interactions across all three cell lines, **(B)** the synergy/antagonism scores for each combination, and **(C)** the synergy scores observed for each cell line individually. MAD = median average deviation of scores across samples.

**Figure 13:**
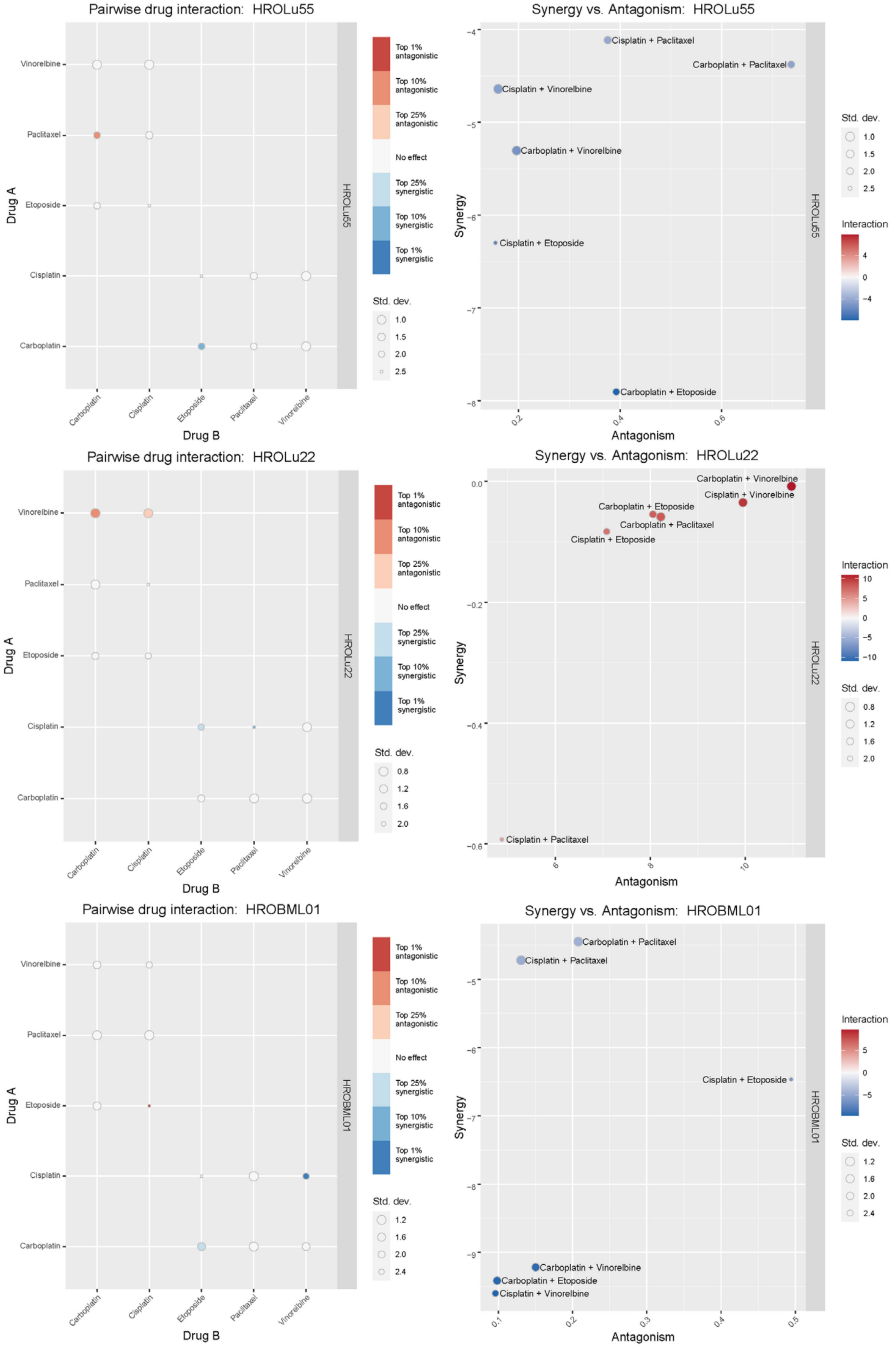
Synergistic effects of drug combinations. The Bliss independence-based synergy scores for HROLu55, HROLu22, and HROBML01 are presented in the graphic.

Treatment of patients with a single substance is rarely performed. Thus, clinically more relevant are drug combinations, which were tested in a so-called checkerboard assay (**Figures 12**, **13** and **Supplementary Data 2** consisting of **Supplementary Table 2** and **Supplementary Figure 1**). Observed interaction scores range from −9.63 (for HROBML01 and the combination of cisplatin with vinorelbine) to 10.95 (for HROLu22 and the combination carboplatin with vinorelbine). Most studies suggest a cutoff at about −10 and 10 (46–48) for calling synergy or antagonism, respectively. None of the tested combinations largely surpass these values; thus, observed effects were most likely additive. More importantly, no antagonistic effects occurred.

## 4 Discussion

Lung cancer is one of the most common tumor entities and accounts, especially in absolute numbers, for many cancer-related deaths, thus underlining the urgency of further research. Precision medicine is on the rise. The individualized therapy selection process and research in this context largely profit from relevant tumor models. We describe three novel PDCs with high similarity to their original tumors and good utility for research in either a basic or translational context. Of special interest is that cell line HROLu55 is derived from the rare subentity of pleomorphic lung tumors. PDC models of this rare subentity are very scarce, especially HROLu55, given the extensive characterization, including WES and RNA-seq analyses. Most importantly, all three cell lines maintained the histological and molecular characteristics of the original patient tumor counterparts.

The cell line HROLu22 represents the adenomatous type, HROBML01 represents the squamous cell type, and HROLu55 represents the pleomorphic lung cancer type. This was confirmed by an expert pathologist (FP). From the generally high level of data concordance, we conclude that these models represent the patients they are derived from very well. This is in line with what we previously already observed for models established from colorectal cancer (8) and glioblastoma (5). These models, in low passages (below passage 30), are thus very useful for general or basic research on the respective subentity, especially HROLu55 for the pleomorphic type. Of note, it was the most invasive (145.4% of control vs. 70.2% and 68.0%) and had the highest migration rate (209.2% of control vs. 90.6% and 70.0%), nearly doubling the values observed for HROBML01 and HROLu22. At the same time, it can be very useful in pre-clinical projects. HROLu55 enables scientists to study mechanisms, functional parameters, and responses of pleomorphic lung tumors. In our dose–response kinetics with drugs commonly used for treatment of lung tumors, no pre-existing resistances were identified and no antagonistic effects were observed for the drug combinations. Although not the focus of this study, we could show in previous investigations that experimentally observed responses (*in vitro* and *in vivo*) corresponded well to the actual clinical outcome of the patients (7). Furthermore, response intensity to classical platinum-based chemotherapeutics of the cell lines and calculated doubling times was weakest for lowest cell proliferation. HROLu22 was least sensitive to cisplatin (IC_50_ of 3.66 vs. 0.73 and 0.50) and carboplatin (IC_50_ of 26.16 vs. 5.66 and 3.62), and at the same time, proliferation was lowest (doubling time of 101.60 h vs. 69.31 h and 72.06 h).

One corner stone of precision medicine is the integration of NGS techniques in the molecular pathological assessments (49). At the UMR, pathological examinations of NSCLC include an Illumina focus panel (for a detailed list of genes included, please see **Supplementary Data 3**). For our small sampling of the three NSCLC patients, we additionally performed WES analyses for the original tumors in comparison to the tumor tissue-derived cell lines. The surprisingly low frequency of mutations identified with the focus panel in routine diagnostics was not confirmed by the high TMB calculated for tumor tissues and PDCs of the WES results. In absolute numbers, the TMB was always higher in the tumor tissue than the corresponding PDC, which, in parts, may be explained by the PDCs consisting of a purer tumor cell population than the tumor tissue. This leads to the comparison of results obtained with 100% pure tumor cells in the PDCs with approximately 50% tumor cells in the tumor tissue. Generally, NSCLC has one of the highest overall TMB with lung adenocarcinoma and lung squamous carcinoma only surpassed by melanoma (40). The discrepancy of focus panel and WES results in our case most likely arises from the observation that most mutations detected in the tumors and PDCs were unique to the tumor tissue and corresponding PDCs. Thus, most likely, many mutations were simply not covered by the focus panel. Also, the EGFR mutation, one of the most common mutations in NSCLC, found in pathological assessment of tumor HROLu22, is present in the WES (raw) data but was discarded in the final report for not passing the variant call quality control filter. The lack of a precisely defined standard for analyzing NGS data, at least on the research level, increases data “variance” enormously and further contributes to our observed discrepancies. Finally, tumor heterogeneity may add to the differences found for tumor tissue and corresponding PDCs since the sample used for DNA isolation and the one used for model establishment might consist of different dominant clones. Additionally, the PDCs undergo further clonal selection simply by *in vitro* culturing processes.

Among the 30 most mutated genes, we discovered familiar cancer candidates such as TP53 and MUC16 (41) and lung cancer associated mutations in the genes MXRA5 (42, 43) and MUC19 (44). The UNC45A mutation detected in the brain metastasis HROBML01 and its corresponding PDCs contributes to tumorigenesis and its expression in cancer cells correlates with proliferation and metastasis of solid tumors (50). In summary, samples of HROLu22 have the lowest numbers of total mutations, samples of HROLu55 have higher mutation rates, and samples from HROBML01 have the highest mutational burden. The TMB for HROLu22 in comparison to the other two samples is even substantially lower. The TBM ratio of approximately 1/10 (HROLu22 vs. HROLu55 and HROBML01) suggests differences in malignant development. Patient HROLu55 is fairly young (48 years) and has a history of (high) nicotine consumption. The tumor of patient HROBML01 metastasized to the brain; thus, accumulation of additional mutations in order to successfully complete the metastasizing process can be assumed. In contrast, the tumor of patient HROLu22 developed without a history of smoking from the fairly old female patient. The cause for mutagenic transformation in this case can only be guessed at. These results are thus not unexpected; they should, however, be interpreted with caution since the lack of normal tissue for HROBML01 may impede correct distinction between germline and somatic mutations.

When it comes to the discovery of new biomarkers and therapeutic targets for personalized medicine, large-scale studies that include multi-omics data are needed, which, in turn, can link genomic and transcriptomic data to phenotypical data. In our small sampling, we observed transcriptomic changes that are currently researched: HOXB9 (Homeobox protein Hox-B9) is the most overexpressed gene across all three of our cell lines. It is often overexpressed in lung cancer, and some studies suggest that overexpression could be linked to promoting invasive properties (51) and small studies in mice have shown a promotion of brain metastases (52). Across the three cell lines tested in this study, HROBML01 has the highest expression followed by HROLu55. A second example of overexpression in our samples is the high-mobility group AT-hook 2 (HMGA2) gene. Overexpression of this gene can be seen in multiple cancer entities and seems to be associated with poor prognosis in lung cancer (53–55). Kita-Kyushu Lung Cancer Antigen-1 (KK-LC-1/CT83) is also highly expressed in our sampling, and the overexpression of this gene was proposed as a target for new precision immunotherapy approaches (56, 57). The observed high expression of ZIC5 is in line with experiments by Sun et al. who could show that ZIC5 is highly upregulated in NSCLC tumor tissues (58), and they suggested that ZIC5 may act as an oncogene by influencing CCNB1 and CDK1 complex expression. Finally ZIC5 is recommended as a biomarker and potential therapeutic target for NSCLC patients (58). Scientists Yang and Liu described that overexpression of BANCR suppresses cell viability and invasion and promotes apoptosis in NSCLC cells *in vitro* and *in vivo* (59). Our observed downregulation thus is, most likely, one of the oncogenic mechanisms exerted by our cell lines. Potential therapeutic effects by BANCR inhibition could be assessed by taking advantage of our PDCs.

In conclusion, we report on three PDCs established from different subentities of NSCLC including the very rare pleomorphic cell type. All PDCs retained morphological, molecular, and genetic properties of their patient tumor tissue originals. Additionally, these PDCs are a 100% pure tumor cell population and, thus, allow, besides functional analyses and response testing *in vitro*, for an easier linking of NGS results to phenotypical tumor characteristics.

## Data availability statement

The raw WES and RNA-Seq Datasets presented in this article are not readily available because they contain confidential information about individuals protected under the General Data Protection Regulation. Requests to access the datasets should be directed to the corresponding author.

## Ethics statement

The studies involving human participants were reviewed and approved by the ethics committee of the University Medical Center Rostock. The patients/participants provided their written informed consent to participate in this study.

## Author contributions

As principal investigator, CL designed the project and supervised experimental execution as well as data analysis. The experiments were performed and obtained data were analyzed by IA. Histological assessments were performed and analyzed by the expert pathologist FP. ML was involved in all project discussions and helped with problem solving. The manuscript was drafted by CL and IA, all authors read and approved the final version of the manuscript.

## In Memoriam

This paper is dedicated to the memory of Christina S. Linnebacher, who passed away unexpectedly while this paper was being peer-reviewed. CSL was an esteemed author whose contributions to the field of oncology research were invaluable. CSL was widely known for her expertise in patient-derived cancer models, which provided crucial insights into the development and treatment of cancer. Her pioneering research will continue to have a profound impact on the scientific community, and she will be deeply missed by her family, friends, and colleagues.
