# Supplementary material for: Establishment, characterization, and drug screening of low-passage patient individual non-small cell lung cancer in vitro models including the rare pleomorphic subentity
Source: Front Oncol. 2023 May 9;13:1089681. doi: 10.3389/fonc.2023.1089681 (PMC10203569; doi:10.3389/fonc.2023.1089681)
Supplement: Supplementary file 1 [file DataSheet_1.pdf]

Supplementary Material

1 Supplementary Data 1:

drug	dose range (HROLu55)	dose range (HROLu22)	dose range (HROBML01)
**Cisplatin**	$5.00x10^{1}$ – $1.22x10^{-2}$ µM	$5.00x10^{1}$ – $1.22x10^{-2}$ µM	$5.00x10^{1}$ – $1.22x10^{-2}$ µM
**Carboplatin**	$4.00x10^{2}$ – $9.77x10^{-2}$ µM	$5.00x10^{2}$ – $9.77x10^{-2}$ µM	$5.00x10^{2}$ – $9.77x10^{-2}$ µM
**Paclitaxel**	$5.00x10^{1}$ - $5.00x10^{-5}$ µM	$5.00x10^{1}$ - $1.56x10^{-5}$ µM	$1.00x10^{2}$- $5.00x10^{-5}$ µM
**Etoposide**	$4.00x10^{2}$ - $1.22x10^{-3}$ µM	$4.00x10^{2}$ - $2.51x10^{-4}$ µM	$4.00x10^{2}$ - $2.51x10^{-4}$ µM
**Vinorelbine**	$1.00x10^{2}$ - $8.19x10^{-8}$ µM	$1.00x10^{2}$ - $8.19x10^{-8}$ µM	$1.00x10^{2}$ - $8.19x10^{-8}$ µM

Supplementary Table 1. Dose range used for determination of single agent IC50 values

2 Supplementary Data 2:

For combination drug testing we used two 6x6 dose response matrices per 96-well plate with one row reserved for untreated control samples and one row for blank controls (Supplementary Figure 1). The dose range was adjusted according to the data obtained from single substance response testing (Supplementary Table 2).

drug	dose range (HROLu55)	dose range (HROLu22)	dose range (HROBML01)
**Cisplatin**	$2.00x10^{0}$ - $1.25x10^{-1}$ µM	$1.40x10^{1}$ - $8.75x10^{-1}$ µM	$2.00x10^{0}$ - $1.25x10^{-1}$ µM
**Carboplatin**	$2.20x10^{1}$ - $1.38x10^{0}$ µM	$1.00x10^{2}$ - $6.25x10^{0}$ µM	$1.60x10^{1}$ - $1.00x10^{0}$ µM
**Paclitaxel**	$8.00x10^{-3}$ - $5.00x10^{-4}$ µM	$1.00x10^{-3}$ - $6.25x10^{-5}$ µM	$8.00x10^{-3}$ - $5.00x10^{-4}$ µM
**Etoposide**	$4.00x10^{-1}$ - $2.50x10^{-2}$ µM	$8.00x10^{-1}$ - $5.00x10^{-2}$ µM	$4.00x10^{-1}$ - $2.50x10^{-2}$ µM
**Vinorelbine**	$1.00x10^{-1}$ - $6.25x10^{-3}$ µM	$1.00x10^{-1}$ - $6.25x10^{-3}$ µM	$1.00x10^{-1}$ - $6.25x10^{-3}$ µM

Supplementary Table 2. Dose range used for drug combination testing

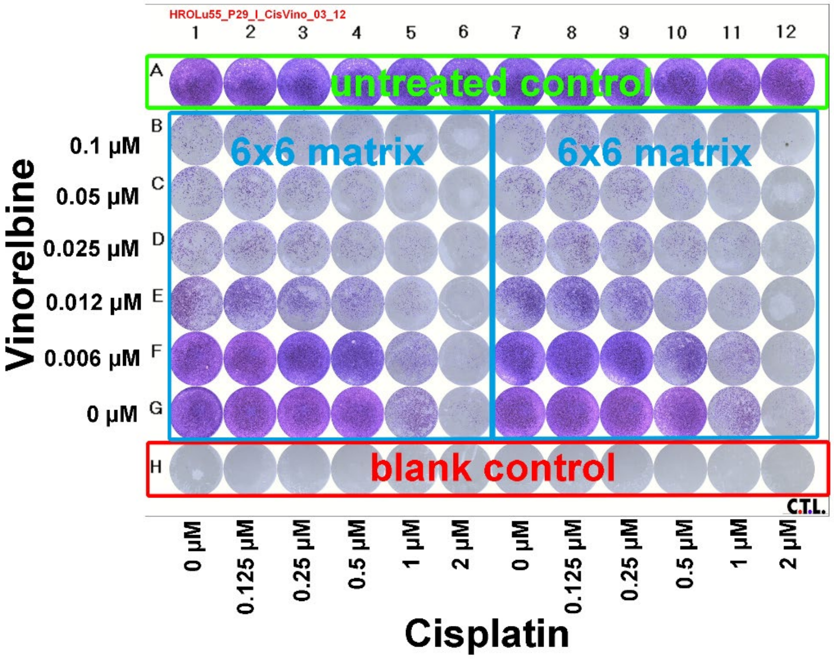

Supplementary Figure 1 Example of 6x6 dose-response matrices format used for drug combination assays.

3 Supplementary Data 3:

Supplementary Table 3 AmpliSeq for Illumina Focus Panel (Illumina, Inc., San Diego, California, U.S.)

**DNA**	**RNA**
AKT1	ABL1
ALK	AKT3
APC	ALK
AR	AXL
BIRC2	BRAF
BRAF	EGFR
BRCA1	ERBB2
CCND1	ERG
CDK4	ETV1
CDK6	ETV4
CTNNB1	ETV5
DCUN1D1	FGFR1
DDR2	FGFR2
EGFR	FGFR3
ERBB2	MET
ERBB3	NTRK1
ERBB4	NTRK2

ESR1	NTRK3
FGFR1	PDGFRA
FGFR2	PPARG
FGFR3	RAF1
FGFR4	RET
GNA11	ROS1
GNAQ	
HRAS	
IDH1	
IDH2	
JAK1	
JAK2	
JAK3	
KIT	
KRAS	
MAP2K1	
MAP2K2	
MED12	
MET	
MTOR	
MYC	
MYCN	
NF1	
NRAS	
PDGFRA	
PIK3CA	
RAF1	
RET	
ROS1	
SMO	

For HROLu22 an addition screening for EGFR mutations was done using the Therascreen EGFR Pyro Kit (Qiagen N.V., Venlo, Netherlands) which is investigating for mutations in codon 719 (exon 18), 768, 790 (exon 20), 858-861 (exon 21), and deletions and complex mutations in exon 19.

4 Supplementary Data 4: RNASeq Principal Component Analysis & Sample-to-sample distances

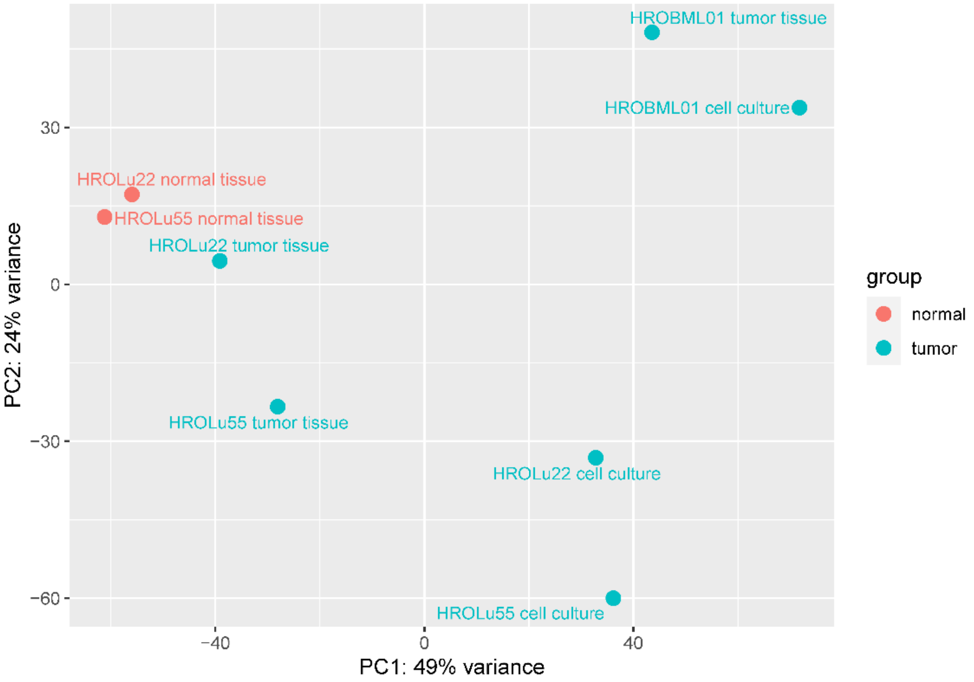

Supplementary Figure 2 Principal component analysis (PCA) plot

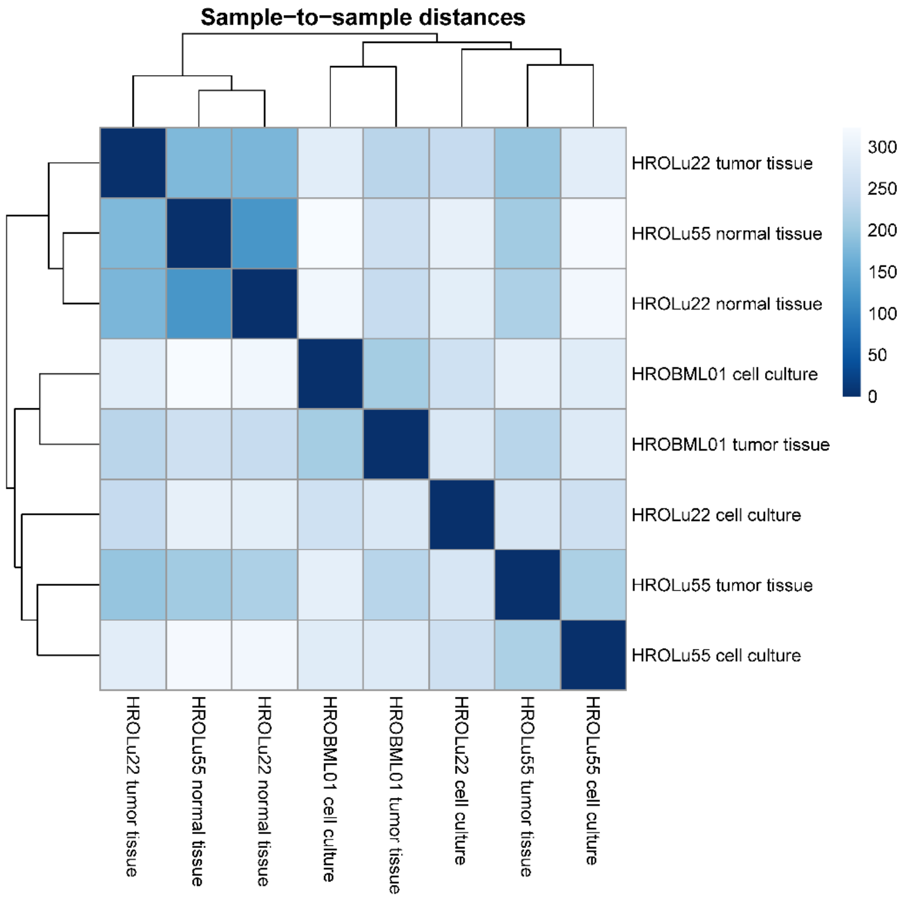

Supplementary Figure 3 Hierarchical clustering heatmap of sample-to-sample distances
